# Dual-Functionalized Nanoliposomes Achieve a Synergistic Chemo-Phototherapeutic Effect

**DOI:** 10.3390/ijms232112817

**Published:** 2022-10-24

**Authors:** Ana Lazaro-Carrillo, Beatriz Rodríguez-Amigo, Margarita Mora, Maria Lluïsa Sagristá, Magdalena Cañete, Santi Nonell, Angeles Villanueva

**Affiliations:** 1 Departamento de Biología, Darwin 2, Universidad Autónoma de Madrid, 28049 Madrid, Spain; 2 Department of Biochemistry and Molecular Biomedicine, Facultat de Biologia, Universitat de Barcelona, 08028 Barcelona, Spain; 3 Institut Químic de Sarrià, Universitat Ramon Llull, Via Augusta 390, 08017 Barcelona, Spain; 4 Instituto Madrileño de Estudios Avanzados en Nanociencia (IMDEA Nanociencia), Faraday 9, 28049 Madrid, Spain

**Keywords:** photodynamic therapy, protoporphyrin IX, irinotecan, chemo-phototherapy, bimodal-functionalized nanoliposomes, synergistic effect, time-lapse microscopy, reactive oxygen species, double-strand DNA break, subcellular location

## Abstract

The enhancement of photodynamic therapy (PDT) effectiveness by combining it with other treatment modalities and improved drug delivery has become an interesting field in cancer research. We have prepared and characterized nanoliposomes containing the chemotherapeutic drug irinotecan (CPT11_lip_), the photodynamic agent protoporphyrin IX (PpIX_lip_), or their combination (CPT11-PpIX_lip_). The effects of individual and bimodal (chemo-phototherapeutic) treatments on HeLa cells have been studied by a combination of biological and photophysical studies. Bimodal treatments show synergistic cytotoxic effects on HeLa cells at relatively low doses of PpIX/PDT and CPT11. Mechanistic cell inactivation studies revealed mitotic catastrophe, apoptosis, and senescence contributions. The enhanced anticancer activity is due to a sustained generation of reactive oxygen species, which increases the number of double-strand DNA breaks. Bimodal chemo-phototherapeutic liposomes may have a very promising future in oncological therapy, potentially allowing a reduction in the CPT11 concentration required to achieve a therapeutic effect and overcoming resistance to individual cancer treatments.

## 1. Introduction

Photodynamic therapy (PDT) is a multi-step and successful clinically approved oncologic therapeutic modality. Photodynamic therapy involves the selective uptake of a photosensitizer (PS) by neoplastic tissue, followed by irradiation with light of a suitable wavelength, able to trigger photochemical reactions that lead to the generation of reactive oxygen species (ROS), mainly singlet oxygen (^1^O_2_), which results in tumor regression. Photodynamic therapy-based antitumor effects are multifactorial and include: (i) direct killing of tumor cells by different cell death mechanisms, (ii) alteration of antiapoptotic factors and proteins responsible for classical drug resistance, sensitizing drug-resistant cells, (iii) damage to tumor vasculature, which decreases tumor growth and enhances vascular permeability improving drug delivery, and (iv) immune response activation against cancer cells. Photodynamic therapy has been approved in several countries to treat a variety of cancers such as skin, bladder, lung, breast, esophagus, and cervix, among others [1].

Compared to other tumor therapies, PDT has several important advantages, especially for superficial lesions: (i) Its direct cytotoxic effect is highly site-specific as PS and light have to converge. (ii) It is a minimally invasive and aggressive procedure, so it does not require general anesthesia. (iii) Its duration is short and no hospitalization is needed. (iv) It does not generate cumulative effects thereby allowing repetitive sessions. (v) Moreover, PDT takes advantage of local tumor control in combination with the immune suppression of distant disease, overcoming also traditional chemoresistance mechanisms. (vi) It has very few side effects, with patients suffering only moderate pain and temporary skin photosensitivity [2,3]. (vii) Furthermore, the excited PS can also be used as a fluorescent probe for photodynamic diagnosis [4]. The ability to accurately define tumor margins is of crucial importance for successful surgical removal [5]. Therefore, PDT may be considered a powerful tool in oncological treatment and bioimaging.

However, this therapy has not achieved the expected success yet, mainly due to (i) the limited light penetration into tissue, although this is being addressed, e.g., by the use of interstitial optical fiber-based light sources [6], and (ii) the development of resistance mechanisms, mainly owing to changes in mitochondria or increased PS efflux [7]. Different strategies are being currently introduced to enhance PDT effectiveness, such as improvement of tumor targeting using nanotechnology, the combination of different PSs, or the combination of PDT with other therapies, thereby potentiating conventional modalities for cancer treatment and decreasing tumor resistance [8,9].

In this line, this study explores the combination of the PS protoporphyrin IX (PpIX) with the chemotherapeutic agent irinotecan (CPT11). PpIX is the active photosensitizing agent of the widely applied drug 5-aminolevulinic acid (5-ALA), approved, among others, by the Food and Drug Administration (FDA) and the European Medicines Agency (EMA) under trade names such as Levulan, Metvix, Ameluz, or Gliolan. 5-ALA shows a high accumulation in malignant cells and a better definition of tumor limits for surgical resection [10,11]. On the other hand, CPT11 is activated by hydrolysis to 7-ethyl-10-hydroxycamptothecin (SN-38), an inhibitor of topoisomerase I, which leads to inhibition of both DNA replication and transcription. It is used for metastatic colorectal and pancreatic cancer treatment, although it shows frequent chemoresistance by P-glycoprotein efflux-based transport [12,13,14].

In our work, both drugs (CPT11 and PpIX) were encapsulated in liposomes, which are the most frequently approved nanovehicles by the FDA and the EMA. Hydrophilic drug molecules such as CPT11 do not easily diffuse through their lipid bilayer, which provides cytoprotection during bloodstream circulation and reduces systemic toxicity. The use of liposomes also alleviates the issue of hydrophobic drug delivery, such as PpIX, releasing the drug inside of the cell when fused with the plasma membrane or when lysed in acidic compartments of the cell (e.g., lysosomes). In addition, as with other nanoparticles, liposomes allow passive tumor targeting by the EPR (enhanced permeability and retention) effect. Therefore, these nanostructures provide both tumor targeting and drug delivery to tumor cells [15,16].

The combination of a PDT agent with a chemotherapeutic drug in a single nanocarrier could enhance the treatment efficiency if the drugs acted synergistically. Moreover, the drugs might then be used at lower doses when compared to the corresponding monotherapies, which could thereby reduce their side effects and avoid resistance mechanisms [17].

In the present study, we investigated a liposome-based drug delivery system (DDS) combining PpIX/PDT with CPT11 for the elimination of HeLa cancer cells and demonstrated that this formulation endows the bimodal treatment with a synergistic effect.

HeLa cells were selected because they are one of the most commonly used cell lines in cancer research. In addition, numerous chemo-phototherapy studies are being performed on this cell line [18,19].

## 2. Results

### 2.1. Preparation and Characterization of Nanoliposomes

CPT11 and PpIX were individually (CPT11_lip_ and PpIX_lip_, respectively) or co-encapsulated (CPT11-PpIX_lip_) in liposomes. The lipid composition was chosen based on the chemical nature of the drugs and previously reported formulations [20,21]. Liposomes were prepared with the ternary lipid mixture *L*-α-distearoyl-phosphatidylcholine (DSPC), *L*-α-dioleoyl-phosphatidylserine (DOPS), and cholesterol (CHOL) (DSPC/DOPS/CHOL, 65/35/30, molar ratio) at a concentration of 10 mg lipid·mL^−1^ by a microemulsification method. In the unimodal liposomes (A), the lipid:drug molar ratio was 7.5:1 in CPT11_lip_ and 150:1 in PpIX_lip_. The same ratios were maintained in the CPT11-PpIX_lip_ bimodal liposomes (B). Values reported are the mean ± SD (standard deviation) of at least three independent experiments.

There are no antecedents of the double encapsulation of both CPT11 and PpIX in liposomes. Initial attempts to encapsulate PpIX, using the lipid mixture mentioned above, showed that a great amount of the photosensitizer was retained in the polycarbonate filters of the extruder device. For this reason, the methodology was adapted to produce liposomes by the microemulsification method. The ratio between PpIX and CPT11 inside bimodal liposomes (CPT11-PpIX_lip_) was established according to the IC50 values found for PpIX_lip_ and CPT11_lip_ (see Figure S1). Physicochemical characteristics for control (blank) liposomes, unimodal irinotecan liposomes (CPT11_lip_), unimodal protoporphyrin IX liposomes (PpIX_lip_), and bimodal irinotecan/protoporphyrin IX liposomes (CPT11-PpIX_lip_) are shown in Table 1.

PpIX was loaded in the lipid bilayer of the liposomes via hydrophobic interactions, while amphiphilic CPT11 was encapsulated mainly in the aqueous core through passive equilibration but also interacting with the negatively charged DOPS. The encapsulation efficiency was greater than 80% for CPT11 and 95% for PpIX, in both unimodal and bimodal liposomes. The molar ratio of the liposome-encapsulated CPT11 and PpIX in CPT11-PpIX_lip_ was kept around 20, which is the molar ratio of their individual IC50 values (see Figure S1). The lipid concentration in all liposomal formulations, around 7.5 mg·mL^−1^, was lower than that of the initial lipid amount used for the preparation of liposomes (10 mg lipid·mL^−1^) due to losses during the microemulsification process and subsequent filtration through the 0.22 μm diameter membrane filters to sterilize the liposomal preparations [15]. Concentrations of CPT11 and PpIX in liposomal suspensions were determined based on their respective absorbance and fluorescence spectra in lactate buffer (pH 4.4) and tetrahydrofuran (THF), respectively, (see Figure S2).

Taking into account that liposomal suspensions are thermodynamically unstable colloidal systems and that liposomes tend to aggregate over time, the stability of CPT11-PpIX_lip_ suspensions was controlled by measuring particle size (see Figure S3), zeta potential (Table 1), and turbidity over time. In this regard, it is interesting to note that the negative ζ-potential of control liposomes does not change with the incorporation of CPT11 and PpIX. The negatively charged surface endows liposomes with greater stability in suspension, avoiding their aggregation owing to electrostatic repulsion [15].

Other relevant details related to the behavior of both drugs either free in solution or loaded in liposomes have been included in the electronic supporting information. Thus, fluorescence emission spectra of CPT11-PpIX_lip_, time-resolved fluorescence of CPT11-PpIX_lip_ suspensions, time-resolved ^1^O_2_ phosphorescence signals of CPT11-PpIX_lip_ are shown in Figures S4–S6, while fluorescence quantum yields and lifetimes of CPT11 and PpIX are collected in Table S1.

### 2.2. Evaluation of Cytotoxicity of Unimodal and Bimodal Nanoliposomes at 24 h after Treatments

**Figure 1:**
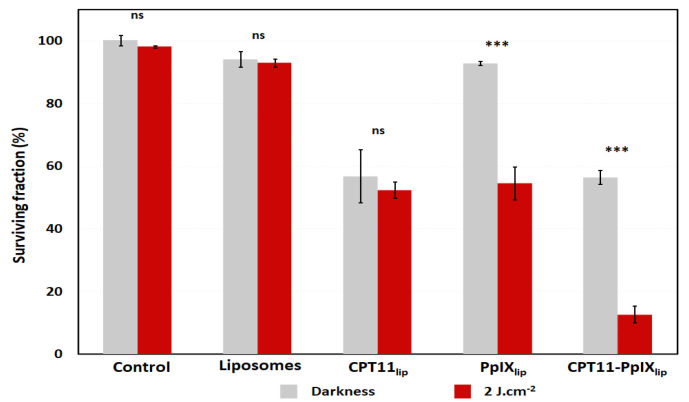
Cell viability of HeLa cells treated with unimodal and bimodal liposomal formulations in darkness and light conditions. Irradiation was performed with a red-light dose of 2 J·cm^−2^. Cell viability was analyzed by MTT assay performed 24 h after each treatment. *p* values < 0.001 (***).

Firstly, we identified the IC50 value of CPT11_lip_ and PpIX_lip_ by the 3-(4,5-dimethylthiazol-2-yl)-2,5-diphenyl tetrazolium bromide (MTT) assay. The IC50, namely the drug concentration that kills 50% of cultured HeLa cells, was used to compare the cytotoxicity induced after treatment with unimodal liposomes (CPT11_lip_ or PpIX_lip_) and a treatment with bimodal liposomes (CPT11-PpIX_lip_). Both formulations were incubated for 24 h and were then exposed to a red-light dose of 2 J·cm^−2^ or kept in the dark for control purposes. The concentrations were the same in irradiated and not irradiated toxicity tests. The MTT tests were performed 24 h after irradiation. The IC50 values for CPT11 and PpIX were 53.3 µM (approx. 50 µM) and 2.8 µM (approx. 2.5 µM), respectively, (Figure S1). Blank liposomes at the same lipid concentration and red-light dose, as well as PpIX_lip_ without subsequent irradiation, did not show a significant cytotoxic effect (Figure 1).

Then, the photocytotoxic effect of the bimodal liposomes was studied. Results of the MTT assay performed 24 h after irradiation showed that incubation with CPT11-PpIX_lip_, at the same concentrations and light dose as in the unimodal treatments, was more effective in the inactivation of HeLa cells (Figure 1), showing a clear synergistic effect, as assessed by Valeriote and Lin’s method (Table S2). The bimodal treatment with CPT11-PpIX_lip_ without light induced a similar effect as CPT11_lip_.

### 2.3. Cell Culture Evolution over Long Periods

**Figure 2:**
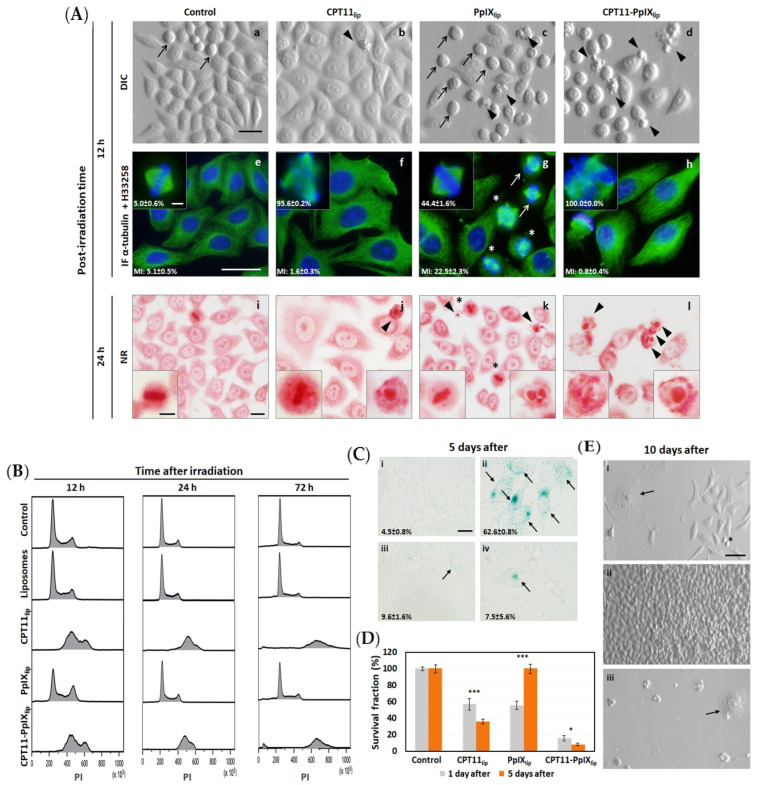
HeLa cell responses at different post-treatment times. (**A**) TOP (**a**–**d**): Treated and non-fixed cells visualized under differential interference contrast (DIC) microscopy after 12 h. MIDDLE (**e**–**h**): Indirect immunofluorescence (IF) for α-tubulin of microtubules (green) and H-33258 counterstaining of DNA (blue) in control or treated cells fixed 12 h after irradiation; the numbers refer to the Mitotic Index (MI) and aberrant mitosis percentage in the same samples. BOTTOM (**i**–**l**): Treated cells were fixed 24 h after irradiation and stained with neutral red (NR). Arrowheads show apoptotic morphologies, arrows mark mitosis and asterisks indicate aberrant mitosis. Bar scale: 25 μm and 5 μm in magnifications in all panels. (**B**) Cell cycle analyzed by flow cytometry revealed by propidium iodide (PI) in photodynamic treated cells at 12, 24, and 72 h after irradiation. (**C**) Effect of photodynamic treatment on HeLa cells five days after irradiation. Analysis of senescent cells by senescence-associated β-galactosidase activity (SA-β-gal) in (**i**) control cells, cells incubated with (**ii**) CPT11_lip_, (**iii**) PpIX_lip_, or (**iv**) CPT11-PpIX_lip_. Arrows show senescent cells. The percentage of positive cells for the SA-β-gal assay relative to the total number of cells (alive plus dead cells) is indicated. Scale bar: 10 µm. (**D**) Cell viability was analyzed by MTT assay performed one or five days after irradiation. (**E**) Cellular morphology of cells incubated with CPT11_lip_ (**i**), PpIX_lip_ (**ii**), or CPT11-PpIX_lip_ (**iii**) visualized directly under differential interference contrast (DIC) microscope ten days after irradiation. The arrow shows senescent cells and the asterisk marks cells undergoing division. Scale bar: 50 μm. *p* values < 0.05 (*) and <0.001 (***).

Next, the possible long-term toxicity induced by the different nanoformulations on HeLa cells was analyzed. Photodynamic treatments of cells using unimodal and bimodal liposomes resulted in different morphological alterations and cell density over time after irradiation (see Figure 2A). 

At 12 h after irradiation CPT-11_lip_ produced an increase in the cellular size, resulting in larger cells (Figure 2A(b)). PpIX_lip_ induced an increase in the number of dividing cells, resulting in round-shaped cells possibly because of a temporary metaphase arrest (Figure 2A(c)). Contrary to CPT-11_lip_, PpIX_lip_ treatments did not produce a cellular size increase. Moreover, after treatment with CPT11-PpIX_lip,_ many cells showed morphological features of apoptosis (cell rounding and shrinkage, and appearance of plasma membrane bubbles).

Metaphase arrest was confirmed by indirect immunofluorescence for α-tubulin, showing also slight alterations in almost half of the metaphases in PpIX_lip_ (some misaligned chromosomes) and completely aberrant cellular divisions in both CPT11 treatments (altered mitotic spindle and chromosome fragmentations and misalignments). In addition, there was a reduced number of mitoses in both treatments with CPT11 (CPT11_lip_ and CPT11-PpIX_lip_) (Figure 2A(e–h)).

Later, at 24 h post-irradiation (Figure 2A(i–l)), the metaphase arrest in PpIX_lip_ was overcome and some late apoptotic morphologies could be seen (Figure 2A(k)). It is important to highlight that CPT11_lip_ does not trigger high levels of cell death, but rather arrests the cell cycle; therefore, the cellular density did not increase as much as in the controls. Similar results have been previously described for free CPT11 (not incorporated into liposomes) [22]. This is the reason why cell viability measured by MTT dropped to 50% compared to control cells. In addition, CPT11_lip_-treated cells doubled or quadrupled in size, and the mitotic index was much lower (compared to untreated cells), which could be a sign of polyploidy. CPT11-PpIX_lip_ showed similar morphological changes, but a high number of cells with chromatin fragmentation could also be observed.

On the other hand, flow cytometry analysis of the cell cycle with propidium iodide DNA staining showed a different response to treatments (see Figure 2B). HeLa cells, incubated with empty liposomes, showed a similar cell cycle to control cells. However, cells treated with CPT11_lip_ led to an increase in DNA content, indicating polyploidization. In addition, at 72 h after treatment a subG_1_ peak appeared after the photodynamic treatment of HeLa cells with bimodal liposomes (CPT11-PpIX_lip_), suggesting a cell death by apoptosis under this condition. Moreover, cells treated with PpIX_lip_ showed a cell cycle arrest at 12 h in the G_2_/M phase, but 24 and 72 h after irradiation the cell cycle profile was similar to the control cells, confirming a normal rate of mitosis.

At 5 days after the different treatments, the β-galactosidase assay was performed to identify a possible induction of senescence. As can be seen in Figure 2C, a significant number of cells treated with CPT11_lip_ (~63%) were positive for this test. In contrast, cells treated with PpIX_lip_ and CPT11-PpIX_lip_ showed much lower values (9.6 and 7.5%, respectively).

To demonstrate that the bimodal liposomes induced progressive cell death, a new MTT assay was performed 5 days after irradiation. Figure 2D shows an increase in dead cells by CPT11-PpIX_lip_ treatment relative to the results of the cytotoxicity assay performed at 24 h.

Finally, at 10 days post-irradiation, the visualization of cell cultures under differential interference contrast (DIC) microscope displayed different responses as a function of treatment received (see Figure 2E). CPT11_lip_-treated cultures showed some large and flattened senescent cells, but regrowth of cells that had survived to the treatment was also detected. Likewise, the cells treated with PpIX_lip_ had proliferated and covered the whole surface of the culture dish. In contrast, CPT11-PpIX_lip_ treatment killed all cells and very few senescent cells were observed 10 days after treatment. but, without any sign of proliferation.

### 2.4. Identification of Apoptotic Cell Death

**Figure 3:**
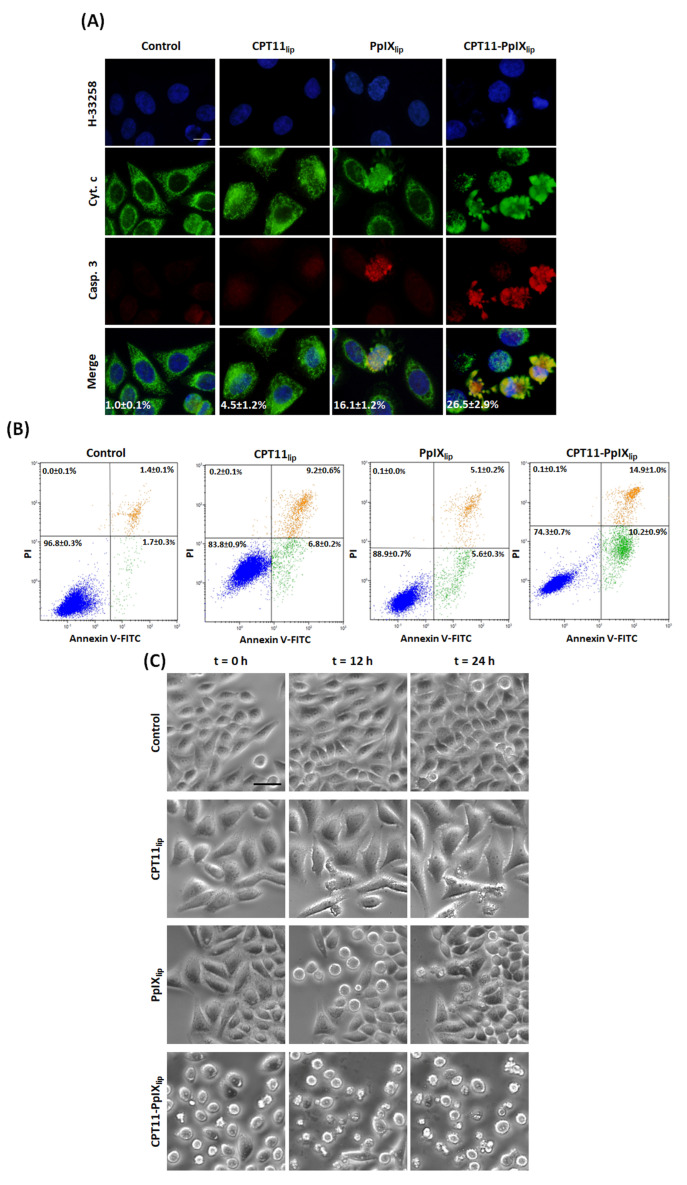
Analysis of apoptotic HeLa cell death. (**A**) Representative images of indirect immunofluorescence for H-33258 counterstaining of DNA (blue), cytochrome c (Cyt. c; green), cleaved caspase-3 (Casp. 3; red), and in control or treated cells 4 h after irradiation. Scale bar: 10 µm. (**B**) Flow cytometry histograms of annexin-V-FITC (Annexin.V-FITC) and propidium iodide (PI) detection in control cells or incubated with CPT11_lip_, PpIX_lip,_ or bimodal liposomes CPT11-PpIX_lip_ 24 h after irradiation. (**C**) Analysis of unimodal and bimodal liposome treatments of HeLa cells by time-lapse microscopy. Video frames of control and treated cells with CPT11_lip_, PpIX_lip,_ or bimodal liposomes at 0, 12, and 24 h after irradiation. Scale bar: 25 µm.

Photodynamic treatments on HeLa cells with unimodal and bimodal liposomes resulted in different patterns of apoptotic cell death, revealed by cytochrome c/cleaved caspase-3 indirect immunofluorescence (DNA counterstaining) (Figure 3A). Cytochrome c release from mitochondria to the cytoplasm, caspase-3 activation and condensed chromatin, at 4 h after irradiation, evidenced the presence of apoptotic cells, mainly after CPT11-PpIX_lip_ treatment. In contrast, those non-apoptotic cells immunostained negatively for cleaved caspase-3 antibody and still showed mitochondria-localized cytochrome c.

Likewise, for longer post-irradiation analysis (24 h), Annexin-V/PI assay by flow cytometry was carried out to evaluate apoptotic cells (see Figure 3B). In addition, in this case, CPT11-PpIX_lip_ treatment led to a higher number of apoptosis than individual treatments.

To corroborate the different effects induced by unimodal and bimodal liposomes, we filmed all these events by time-lapse microscopy performed from 0 to 24 h after irradiation (see Electronic Supporting Information for a complete video of control and treated cells, Supplementary Videos S1–S4). As can be seen in the selected images in Figure 3C, we were able to corroborate the results described above such as the increased cell size over time by CPT11_lip_, the metaphase arrest at 12 h after irradiation by PpIX_lip_ that was overpassed at 24 h, and frequent apoptosis induced by CPT11-PpIX_lip_.

### 2.5. Subcellular Location

**Figure 4:**
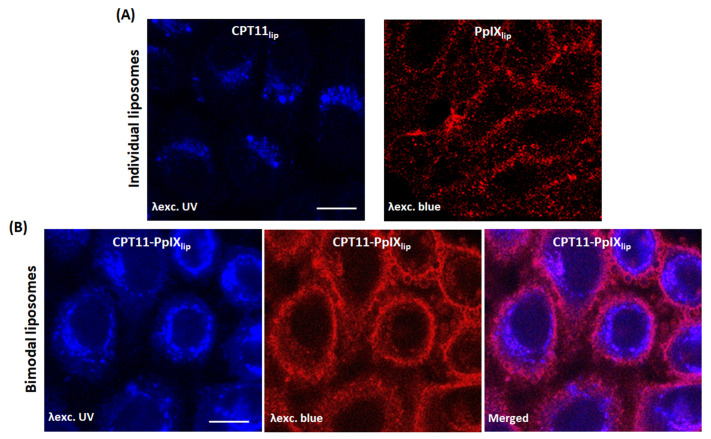
Subcellular location of CPT11 and PpIX on HeLa cells. (**A**) Representative images by confocal microscopy of drug location into cells incubated with the individual (TOP) or (**B**) bimodal liposomes (BOTTOM). Scale bar: 10 μm.

The intracellular location of both drugs in HeLa cells after 24 h incubation with the different liposomal formulations was studied by confocal microscopy (see Figure 4). For CPT11_lip_ the fluorescent signal was visualized mainly in dots in the cytoplasm, which is likely to correspond to CPT11 encapsulated into liposomes delivered to lysosomes. On the other hand, cells incubated with PpIX_lip_ showed a red fluorescence emitted from well-defined spots in the cytoplasm, particularly strong in those areas where two cellular membranes, from two adjacent cells, were in contact. After incubation with CPT11-PpIX_lip,_ most of the irinotecan was localized in lysosomes, but additionally, a diffuse blue emission was seen in the whole cell, including the nucleus, under UV excitation. Likewise, the emission from PpIX showed a distribution pattern similar to PpIX_lip_. Interestingly, the superposition of fluorescence images shows a partial co-localization of CPT-11 and PpIX, with a pink emission, especially at the level of the plasma membrane.

### 2.6. Action Mechanisms of the Different Photodynamic Treatments

**Figure 5:**
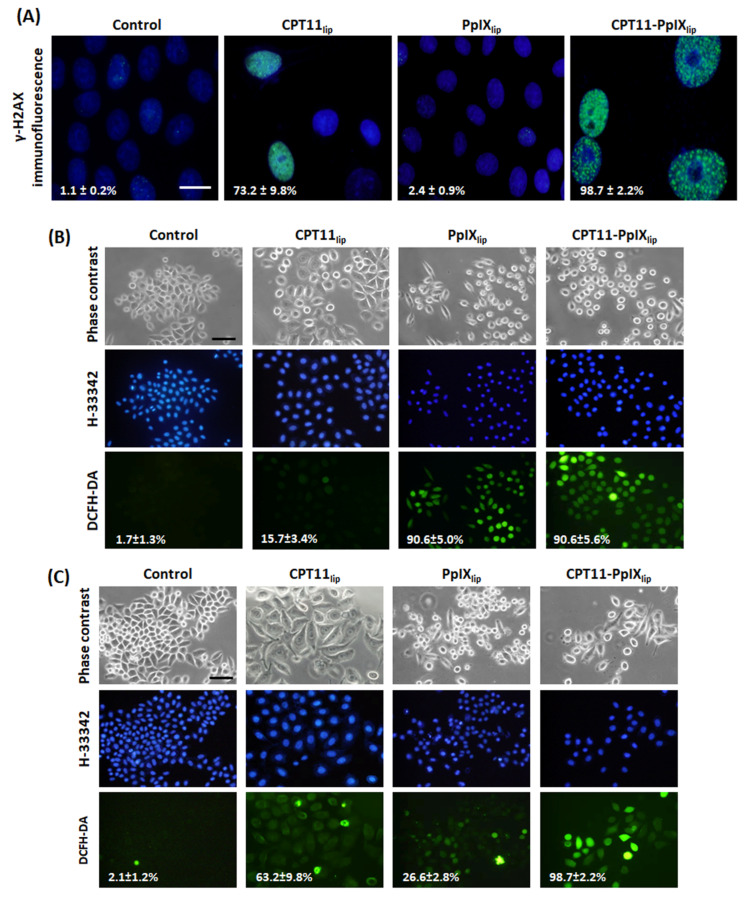
Action mechanisms of the different photodynamic treatments. (**A**) Representative images of indirect immunofluorescence for γ-H2AX (green) and H-33258 counterstaining of DNA (blue) in control or photodynamic treated cells 72 h after irradiation and the percentage of cells with double-strand DNA breaks. Scale bar: 20 µm. (**B**,**C**) Representative images of reactive oxygen species (ROS) generation in control cells and cells submitted to the different photodynamic treatments, analyzed by the DCFH-DA probe 2 h (**B**) and 24 h (**C**) after irradiation. Cells were visualized under phase contrast microscopy or fluorescence microscopy for the DCFH-DA probe (green). Scale bar: 50 µm.

Finally, we aimed to study the main causes of the synergistic effect of the bimodal treatment. As can be judged from Figure 5A, CPT11-PpIX_lip_ treatments induced the most genotoxic damage to HeLa cells. In fact, at 72 h after treatment, practically all the cells in the culture (98.7%) were positive for immunofluorescence for γ-H2AX (a marker of DNA double-strand breaks). This percentage was reduced to 73.2% in the treatment with CPT11_lip_ and 2.4% with PpIX_lip_. These results indicated that CPT11 was able to be released from the liposomes where it was delivered and was able to reach the cell nucleus where it performed its cytotoxic action.

To further understand the mechanisms of action of the different liposomes, an analysis with the 2,7-dichlorodihydrofluorescein diacetate (DCFH-DA) probe for the detection of ROS was also carried out (see Figure 5B,C). At 2 h after irradiation, a similar level of ROS generation using either PpIX_lip_ or CPT11-PpIX_lip_, as well as a much lower but non-zero level with CPT11_lip_, was observed. However, 24 h after irradiation, the ROS level increased in the case of CPT11_lip_, decreased for PpIX_lip_, and virtually all cells treated with CPT11-PpIX_lip_ were labeled for the ROS assay.

All these results indicate that chemo-phototherapeutic treatments with CPT11-PpIX_lip_ are more effective in killing HeLa cells than individual treatments since they can induce both DNA double-strand breaks and ROS in practically all cells.

## 3. Discussion

The use of nanotechnology for the delivery of chemotherapeutic drugs has undergone a spectacular development in recent years for the treatment of cancer, to obtain safer and more effective alternative treatments, as has been reported in recent reviews [23,24,25,26,27].

In this sense, liposomes are a type of nanoparticle with a promising future for their application in the treatment of neoplastic diseases [28,29].

Several chemotherapeutic drugs formulated in liposomes have already been approved by the FDA for the treatment of patients with different types of cancer, including doxorubicin (Doxil^®^) [30] and irinotecan, also known as pegylated liposomal irinotecan (Onivyde^®^) [31], among others.

On the other hand, combination therapies for more than one approved chemotherapeutic drug, using nanocarriers as the drug delivery systems, are being specially designed to achieve greater therapeutic efficacy and other important goals, such as decreasing the doses of the drugs used to reduce unwanted side effects and also to prevent the development of chemoresistance [32].

In recent years, a new strategy in cancer treatment is also being explored, consisting in combining chemotherapy with other oncological therapeutic modalities, such as photodynamic therapy (PDT), photothermal therapy (PTT), and immunotherapy [33,34].

In particular, the combined therapy by co-encapsulation in the same nanoparticle (liposomes included) of a photodynamically acting photosensitizer and a chemotherapeutic drug has received special attention, in so-called Photochemotherapy or Chemophototherapy, due to the synergistic cytotoxic effects that have been described [35,36,37,38,39,40,41].

Here, we designed chemophototherapeutic liposomes which demonstrated their efficacy to kill tumor HeLa cells by combination therapy with two clinically approved drugs, PpIX as a photosensitizer in PDT [42] and CPT11 (irinotecan), a camptothecin derivative widely used in the treatment of colorectal and pancreatic cancer [43]. We hypothesized that by combining a photodynamic photosensitizer (PpIX) with a chemotherapeutic drug (CPT11) in the same liposome, the therapeutic efficacy could be improved.

We first evaluated the ability to kill HeLa cells by administering the drugs individually in liposomes, and comparing their effect when a liposome carried both compounds. The results obtained by colorimetric MTT assay evidenced that the bimodal liposomes (CPT11-PpIX_lip_) had a synergistic cytotoxic effect when compared to the individual treatments, and only about 10% of the HeLa cells were able to survive the chemophototherapeutic treatment.

In this regard, it has recently been demonstrated that different chemophotodynamic treatments can induce a synergistic cytotoxic effect on HeLa cells [41,44,45,46,47,48,49].

However, to our knowledge, this is the first time that liposomes co-delivering PpIX and irinotecan have been assayed. On the nearest line, Huang et al. described important effects on MIA PaCa-2 human pancreatic cell line with benzoporphyrin derivative BPD (Visudyne®) and irinotecan, but incorporated in individual nanoliposomal formulations [50].

On the other hand, the progressive evaluation of the effects induced by the combined treatment at increasingly longer times after treatment completion provided valuable mechanistic insight (see Figure 2). Such studies over time after the end of treatment are infrequently conducted in most research, but they provide valuable information.

At short times, PpIX_lip_ treatment induced a temporary mitotic arrest, confirmed by immunofluorescence techniques for α-tubulin and cell cycle analysis, with a subsequent apoptotic death induction. Both effects vanished over time and the remaining cells that survived proliferated again. In this sense, induction of cell cycle blockage at metaphase-anaphase transition with multipolar spindles has also been described in HeLa cells by PDT with Methyl-aminolevulinate (a PpIX precursor intermediate of heme synthesis), which trigger cell death by apoptosis [51]. In contrast, CPT11_lip_ treatments showed longer-term effects, including polyploidization and the onset of senescence morphologies, but regrowth of the cell culture was also detected 10 days after treatment. In this regard, Haug et al. [52] have also described that CPT-11 administered in its free form (not in some type of nanoparticle) does not produce HCT116 (human colon cancer cell line) cell death until 96 h after treatment. On the other hand, CPT11-PpIX_lip_-treated cells exhibited a mixture of PpIX_lip_ and CPT11_lip_ cellular responses. Significantly, this dual treatment was able to trigger HeLa cell death without showing any signs of regrowth at longer post-treatment times (10 days). Furthermore, using different techniques (immunofluorescence for both cytochrome c and cleaved caspase 3, Annexin V-IP assay, and time-lapse videomicroscopy), we were able to confirm that treatment with CPT11-PpIXlip induced greater cell death by apoptosis than mono-therapy liposomes. In this regard, it is important to highlight the importance of apoptosis as a target for cancer therapy [53,54,55].

Taking advantage of the fluorescent properties of PpIX and CPT11, we also identified their subcellular localization when delivered individually (CPT11lip and PpIXlip) or combined CPT11-PpIX_lip_, using confocal microscopy. Previously, we had described that “free” PpIX accumulates at the plasma membrane of HeLa cells [56]. On the contrary, as can be seen in Figure 4, after HeLa cells were incubated with PpIXlip, an intense red fluorescence was detected not only at the plasma membrane but also in the cytosol in the form of small rounded granules. On the other hand, CPT11lip was mainly localized into lysosomes. Lysosomal localization of CPT11 was previously described by our group using a liposomal formulation [21]. When HeLa cells were incubated with the bimodal CPT11-PpIX_lip_, a substantial fraction of the red fluorescence from PpIX co-localized with the blue fluorescence of CPT11, as shown by the pink color of the overlaid fluorescence. However, a weak blue fluorescence distributed throughout the cell, including the nucleus, was also observed. This would mean that a fraction of the PpIX and CPT11 molecules would have been released from the liposome and diffused throughout the cytosol and even the nucleus, in the case of CPT11.

These results are of key relevance to the present research and are, in part, similar to those described by Zhu et al. [35]. These authors proposed the design of PpIX-loaded liposomes as optimal carriers for chemotherapeutic drugs (in their case, doxorubicin or cisplatin) because they achieve very important advantages: (i) they facilitate the release of the encapsulated drug because when PpIX/Dox bimodal liposomes reach the surface of cancer cells, the PpIX molecules spontaneously leave the liposome membrane and insert into the plasma membrane of the cancer cell, leading to the release of encapsulated Dox (or cisplatin), (ii) drug resistance can be reversed after irradiation because activated PpIX can destroy the efflux pump system, thereby facilitating the entry of the drug into the nucleus; (iii) they can induce a higher cytotoxic effect on tumor cells since, to the action of the drug, the photocytotoxic effect of PpIX accumulated in the membrane is added. It is well known that photosensitizers that accumulate in the plasma membrane are effective in photodynamic treatments [57,58,59].

The increased cytotoxicity of CPT11-PpIX_lip_ could also be partly produced by a photochemical internalization (PCI) mechanism, which involves the use of vesicles containing a photosensitizer, in our case PpIX, whereby the liposomes would be endocytosed by cells and accumulated in lysosomes. Photoactivation of the photosensitizer may cause the rupture of these vesicles allowing CPT11 to escape into the cytoplasm or the nucleus instead of being sequestered there or degraded by its hydrolytic enzymes [60].

Furthermore, we verified that internalized CPT11 was able to induce genotoxic damage in cells incubated with either CPT11_lip_ or CPT11-PpIX_lip_. Virtually all cells in the culture were positive for γ-H2AX immunofluorescence, a marker of DNA double-strand breaks (DSB). It is well known that topoisomerase I inhibitors, such as irinotecan, are capable of inducing DSB that ultimately leads to cell death [61]. However, liposomes carrying only PpIX did not induce DSB. To our knowledge, there is nothing in the literature indicating that PpIX is able to produce DSB.

It should be noted that the synergistic cytotoxic effect achieved was probably due to the higher and persistent level of ROS generation (evaluated by the DCFH-DA probe) after the bimodal treatment when compared to the single treatments. It is well known that both CPT11 and PpIX-PDT are capable of generating ROS individually [62,63,64]; therefore, the simultaneous application of both treatments may well lead to an enhanced ROS production, finally leading to irreparable cell lesions, just as it has been observed in other chemotherapeutic combinations [65]. Increasing evidence demonstrates that intracellular ROS generation is closely connected with apoptosis [66,67,68]. However, it is interesting to note that recent research has shown that the generation of ROS plays a dual role in being able to generate the death of tumor cells, but also mediating redox signaling events beneficial for the progression of the disease [69,70,71].

## 4. Materials and Methods

### 4.1. Materials

L-〈-Distearoyl-phosphatidylcholine (DSPC), L-〈-Dioleoyl-phosphatidylserine (DOPS), and cholesterol (CHOL) were purchased from Avanti Polar Lipids (Birmingham, AL, USA). Irinotecan (CPT11), purchased from Afine Chemicals Limited (Hangzhou, China), was pure with a minimum grade of 99%. Protoporphyrin IX (PpIX), purchased from Frontier Scientific (Logan, UT, USA), had a minimum purity of 99% and was used as received. 3-(4,5-Dimethylthiazol-2-yl)-2,5-diphenyl tetrazolium bromide (MTT) was purchased from Sigma Aldrich (Saint Louis, MO, USA). All other chemicals were commercially available reagents of at least analytical grade. Milli-Q water (Millipore Bedford, Massachusetts system, resistivity of 18 MΩ cm) was used.

### 4.2. Cell Cultures

Human cervix adenocarcinoma HeLa cells ATCC CCL-2^TM^ were obtained from American Type Culture Collection (ATCC, Manassas, VA, USA). Cells were grown as monolayer cultures in Dulbecco’s Modified Eagle’s Medium (DMEM) supplemented with 10% (*v*/*v*) fetal bovine serum (FBS), 50 U mL^−1^ penicillin, and 50 μg mL^−1^ streptomycin (whole medium). All products were purchased from Thermo Fisher Scientific (Waltham, MA, USA) and sterilized through 0.22 μm filters (Merck; Darmstadt, Germany). Cell cultures were maintained in a SteriCult 200 incubator (Hucoa-Erloss; Madrid, Spain) with a 5% CO_2_ atmosphere at 37 °C. Depending on the protocol, cells were seeded in 25 cm^2^ surface flasks (F25) or 24-well plates with or without 10 mm square coverslips (Menzel-Gläser; Brunswich, Germany). All sterile plastics were obtained from Corning (Corning, NY, USA). Treatments were carried out 3 days after seeding at a cellular density of 3000 cells/cm^2^, when cells were in the exponential growth phase, with approximately 60–70% confluence.

### 4.3. Preparation of Liposomes

Intermediate unilamellar liposomes (IUVs) were prepared by the microemulsification method, using a ternary mixture of DSPC:DOPS:CHOL in a molar ratio of 65:35:30, as reported previously [20,21]. The mixture of lipids was dissolved in the minimum amount of chloroform, together with a small amount of methanol to obtain a clear solution. The organic solvents were evaporated by rotary evaporation yielding a thin lipid film on the sides of a round bottom flask and the lipid film was hydrated with 10 mM (pH 4.4) lactate buffer to a final lipid concentration of 10 mg·mL^−1^, to obtain control blank liposomes (i.e., liposomes without encapsulated drugs). The bimodal liposomes were obtained by adding the appropriate amount of CPT11 and PpIX to the chloroform ternary lipid mixture, maintaining the lipid:drug molar ratio at 7.5:1 or 150:1, respectively. The MLV dispersions were microemulsified (EmulsiFlex B3 device, Avestin, Ottawa, ON, Canada) by pumping the fluid 10 cycles through the interaction chamber at 110 kPa. The unimodal liposome counterparts (with CPT11 alone or PpIX alone) were prepared in the same way by adding to the chloroform ternary lipid mixture CPT11 or PpIX. All liposome preparations were sterilized by filtration with a 0.22 μm diameter filter and stored in the dark at 4 °C.

Unimodal liposomes containing CPT11 or PpIX were named CPT11_lip_ or PpIX_lip_, respectively. The bimodal liposomes, containing both drugs at the same time, were identified as CPT11-PpIX_lip_.

### 4.4. Characterization of Liposomes

Zetasizer Nano-ZS (Malvern Instruments Ltd., Worcestershire, UK) was used to measure particle size and zeta potential. Concentrations of total PpIX and CPT11 were quantified spectrophotometrically in THF by using calibration curves under the same conditions. The fraction of non-encapsulated CPT11 was determined by filtration and centrifugation with Centricon YM-10 Filter Devices (Millipore Corporation, Bedford, MA, USA) and quantified by absorption spectroscopy in lactate buffer (pH 4.4). The absorbance was measured at 369 nm and compared with a CPT11 calibration curve (0.002–0.01 mg CPT11·mL^−1^). On the other hand, the non-encapsulated PpIX fraction was determined by centrifugation since it forms aggregates in the external aqueous buffer and precipitates. Once the supernatant was carefully removed, the pellet was resuspended and quantified using fluorescence spectroscopy in THF. The fluorescence was measured using a λ_exc_ of 504 nm and a λ_em_ of 632 nm and compared with a PpIX calibration curve (0.0001–0.001 mg PpIX·mL^−1^).

The stability of liposomes during storage was controlled by measuring their size and zeta potential over time. In addition, absorbance spectra of liposomal suspensions were collected to monitor whether any turbidity or scattering increased over storage time.

The lipid content in the liposome suspensions was measured following Stewart’s method [72], reading the absorbance at 465 nm by comparison with a calibration curve.

Drug entrapment efficiency is defined as the mass ratio of drug entrapped into nanoliposomes to the total drug added initially (DEE %). Drug loading efficiency is determined by the ratio of final drug weight to the overall weight of lipids used to procure the liposomes (DLE %).

### 4.5. Photodynamic Treatments

Cells were incubated for 24 h with CPT11_lip_, PpIX_lip_, or CPT11-PpIX_lip_ maintaining the same drug concentration (50 μM CPT11 and 2.5 μM PpIX) in whole culture medium. After 24 h, incubation cells were washed three times with DMEM and maintained in whole medium during irradiation and post-treatment time. Irradiation was performed by a red light-emitting diode (LED) device (Foco Par 64, Showtec; Shoreham, UK) (λ_max_ = 622 ± 20 nm) with a fluence rate of 7 mW·cm^−2^ during 4.75 min to administer a total light dose of 2 J·cm^−2^ according to the equation:

Total light dose (J·cm^−2^) = Fluence rate (W·cm^−2^) × Irradiation time (s).

Either immediately or at different times after irradiation, different methodological protocols were performed. Besides, experiments were done by incubation with the corresponding blank (empty liposomes) or the loaded liposomes without irradiation (dark toxicity), to examine possible cytotoxic effects exercised by the lipid or the drugs.

### 4.6. 3-(4,5-Dimethylthiazol-2-yl)-2,5-diphenyl Tetrazolium Bromide (MTT) Assay

Phototoxicity was evaluated 24 h after the photodynamic treatment by the MTT dye reduction colorimetric assay, performed as previously [73]. For dark toxicity, cells were incubated with the drugs at the same time but omitting the irradiation process.

### 4.7. Living Cells Imaging by Differential Interference Contrast Microscopy

HeLa cells were grown and directly observed at different post-irradiation times (from 0 to 10 days) by differential interference contrast (DIC) microscopy, as previously [74]. This allows us to see real-time morphological changes such as rounding up of cells, formation of apoptotic bodies, cellular size increase, or cell growth after the different treatments.

### 4.8. Neutral Red Staining

The general cellular morphology and the chromatin state were analyzed by neutral red (NR) staining. For this protocol, cells grown on coverslips, after the different treatments, were washed with PBS, fixed with methanol at −20 °C for 5 min, and air-dried. They were then stained with a 0.1% NR solution in distilled water for 2 min. After removing the dye excess with distilled water, the samples were allowed to air dry and mounted on microscope slides (Thermo Fisher Scientific, Waltham, MA, USA) with the definitive hydrophobic mounting medium DePeX (Serva; Heidelberg, Germany).

### 4.9. Time-Lapse Microscopy

Cells seeded in chambered coverslips (Ibidi; Martinsried, Germany) were recorded by video-microscopy after treatment. Frames were acquired by phase contrast microscopy every 15 min from 0 to 24 h after irradiation, maintaining CO_2_, temperature, and humidity conditions in the cell culture range.

### 4.10. Indirect Immunofluorescence for α-Tubulin, Cytochrome c, Cleaved Caspase-3, and γ-H2AX

All immunofluorescences were performed according to [75], with the only exception that, in α-tubulin immunofluorescence, cells were fixed 5 min in methanol at −20 °C for better microtubule preservation. The DNA breaks were analyzed by γ-H2AX as previously [76].

### 4.11. Annexin-V and Propidium Iodide Analysis by Flow Cytometry

The contribution of apoptosis to the cell death mechanism after the different treatments was also evaluated by flow cytometry with ApoScreen™ Annexin-V apoptosis detection kit conjugated with FITC of Southern Biotech (Birmingham, AL, USA) (AnnexinV-FITC) in combination with the DNA intercalating agent propidium iodide (PI) (kit DNA-Prep Reagents 6607055, Beckman-Coulter; Brea, CA, USA).

After the photodynamic treatments, HeLa cells seeded in F25 flasks were evaluated by this assay at 24 h post-irradiation. To this end, supernatant with detached cells was collected and cells still adhered were trypsinized (Thermo Fisher Scientific, Waltham, MA, USA), added to the previously collected ones, and centrifuged for 4 min at 300× *g* (JP Select; Abrera, Spain). The supernatant was removed, and the pellet was resuspended with DMEM without phenol red (Thermo Fisher Scientific, Waltham, MA, USA). After two washes with PBS at 4 °C, cells were counted by a hemocytometer and 10^6^ cells were resuspended in 100 μL of Annexin binding buffer from the kit diluted 1:10 in distilled water. Subsequently, 10 μL of AnnexinV-FITC was added to the samples, shaken gently with a vortex, and incubated for 15 min on ice protected from light. After incubation, 380 μL of binding buffer diluted 1:10 was added as well as 10 μL of PI at a concentration of 50 μg·mL^−1^, just before measurement by cytometer. A flow cytometer Gallios (Beckman Coulter Inc, Brea, CA, USA) with 488 nm excitation laser and 525/40 filter was used to detect the AnnexV-FITC and 620/30 filter for PI.

### 4.12. Cell Cycle Analysis by Flow Cytometry

The cell cycle of photodynamic treated cells grown in F25 flasks was evaluated at different post-irradiation times (12, 24, and 72 h), as previously described [77].

### 4.13. Senescence-Associated β-Galactosidase Assay

Cells were seeded on coverslips, incubated with the different liposomal formulations for 24 h, irradiated by a red light-emitting diode at a total light dose of 2 J·cm^−2^ and analyzed after 5 days by senescence β-galactosidase staining kit (CS0030, Merck, Kenilworth, NJ, USA) following instructions of the manufacturer.

### 4.14. Reactive Oxygen Species Detection by DCFH-DA Probe

Intracellular levels of reactive oxygen species (ROS) were qualitatively measured using the 2,7-dichlorodihydrofluorescein diacetate (DCFH-DA) fluorescence probe. Cells were grown on coverslips, submitted to photodynamic treatment, and analyzed 24 h after irradiation. Cells incubated for 1 h 30 min with 800 mM H_2_O_2_ in whole medium were used as a positive control of the experiment, leading to ROS production 3 h after the incubation. In all cases, the samples were washed with DMEM and incubated with the DCFH-DA probe (Merck) at a concentration of 10 μM for 30 min. Then, samples were washed in medium without phenol red and immediately visualized in bright field or fluorescence optical microscopy.

### 4.15. Subcellular Localization of CPT11 and PpIX

CPT11 and PpIX accumulation into HeLa cells was localized by confocal microscopy. HeLa cells seeded in plates with coverslips were incubated for 24 h with unimodal or bimodal liposomal formulations at the concentrations mentioned previously. After incubation, cells were washed twice with cell culture medium without phenol red and observed directly by confocal microscopy.

### 4.16. Microscopy and Settings Microscope

Observations of samples processed for optical microscopy (bright field and fluorescence) were made with an Olympus BX61 epifluorescence microscope equipped with an Olympus DP50 digital camera (Olympus; Center Valley, PA, USA). The following filters were used to visualize the fluorescent signal of the fluorophores: UV (365–390 nm) for Hoechst 33258 and 33342, blue (460–490 nm) for Alexa Fluor 488 and DCFH-DA, and green (510–550 nm) for Alexa Fluor 555. The visualization of the location of liposomes with CPT11 and PpIX was performed with a Leica TCS SP8 confocal microscope (Leica Microsystems, Wetzlar, Germany), using a 405 nm laser and collecting the signal with a spectral detector of 413–500 nm for the CPT11 and exciting with a 500 nm laser and picking up the signal at 622–715 nm for the PpIX. In addition, living cells were imaged under a differential interference contrast (DIC) inverted microscope (Leica DM IL LED) equipped with a Leica DFC420 C digital camera. Finally, video-microscopy was carried out with a Leica DMI 6000B inverted microscope with an OrcaR2 camera (Hamamatsu Photonics; Hamamatsu, Japan) and an incubator system with CO_2_ and temperature controller (Okolab; Ottaviano, Italy).

Images were processed using the Adobe Photoshop 7.0 software (Adobe Systems; San José, CA, USA) or ImageJ (Fiji image processing package).

### 4.17. Statistical Analysis

Results corresponded to mean values ± standard deviation from at least five different experiments. For statistical calculations, one-way ANOVA Tukey’s test and GraphPad Prism 5 software (GraphPad Inc.; La Jolla, CA, USA) were used. *p* values < 0.05 (*), <0.01 (**), and <0.001 (***) were considered as statistically significant.

The analysis of the effect of the bimodal liposomal photodynamic treatment was evaluated according to the method described by Valeriote and Lin [78]. [A] represents the cell viability for the unimodal photodynamic treatment with CPT11_lip_, [B] the cell viability for the unimodal photodynamic treatment with PpIX_lip,_ and [A + B] the cell viability for the bimodal photodynamic treatment with both drugs (CPT11-PpIX_lip_). The effect of the bimodal photodynamic treatment is defined as: synergistic if [A + B] < [A] × [B]; additive if [A + B] = [A] × [B]/100; subadditive if [A] × [B]/100 < [A + B] < [A], when [A] < [B]; interference if [A] < [A + B] < [B], when [A] < [B]; and antagonistic if [B] < [A + B], when [A] < [B].

## 5. Conclusions

Our initial experiments investigating the effects of bimodal photodynamic treatment with CPT11-PpIX_lip_ on cell viability showed synergistic cytotoxic effects on HeLa cells, with relatively low doses of PpIX/PDT in combination with CPT11. Subsequently, we analyzed in detail the cellular inactivation mechanisms involved in the bimodal treatment, revealing apoptosis, mitotic catastrophe, and senescence. We found that PpIX/PDT in combination with CPT11 could enhance anticancer activity through a sustained ROS generation, which increases the number of double-strand DNA breaks. In addition, both selected drugs showed fluorescent properties, even when encapsulated. Overall, these bifunctionalized liposomes provide an experimental proof of concept for the clinical application of this liposomal formulation.

Taken together, the results presented in this paper demonstrate a synergistic anticancer activity of PpIX/PDT when co-delivered with CPT11 in liposomes and provide a mechanistic understanding of the synergistic effect. Translation of these results to the clinic might allow a reduction of the CPT11 concentration required to achieve a therapeutic effect, thereby reducing its side effects and the onset of resistance. Finally, we note that the fluorescence properties of both drugs might also allow their application in bioimaging to monitor tumor location and growth, as well as treatment efficacy in real time.

In summary, bimodal chemo-phototherapeutic liposomes have a very promising future in oncological therapy, potentially overcoming anticancer drug resistance and serving as bioimaging agents.

## Figures and Tables

**Table 1:** Physicochemical characterization of liposomes.

Encapsulated Drug		Z-Ave (nm) ^1^	PI ^2^	ζ-Pot (mV) ^3^	DLE % (*w*/*w*) ^4^	DEE % (*w*/*w*) ^5^	[CPT11] (mM) ^6^	[PpIX] (µM) ^6^	[Lipid] (mg·mL^−1^)
Blank		96 ± 12	0.23 ± 0.04	−59 ± 4	-	-	-	-	7.3 ± 0.2
**A**	CPT11_lip_	189 ± 17	0.19 ± 0.10	−58 ± 7	12.5 ± 5.1	82 ± 9	1.26 ± 0.23	-	6.8 ± 0.6
	PpIX_lip_	93 ± 10	0.23 ± 0.03	−59 ± 6	0.5 ± 0.1	98 ± 3	-	65 ± 14	7.2 ± 0.5
**B**	CPT11_lip_	169 ± 18	0.19 ± 0.03	−57 ± 3	11.1 ± 1	85 ± 7	1.21 ± 0.09	-	7.5 ± 0.3
	PpIX_lip_				0.5 ± 0.1	89 ± 7	-	63 ± 9	

^1^ Particle size measured as Z average mean. ^2^ Polydispersity Index. ^3^ Zeta-potential. ^4^ Bulk encapsulated drug concentration in the liposome suspension. ^5^ Drug encapsulation efficiency expressed as a drug weight percentage (*w*/*w*). ^6^ Bulk encapsulated drug concentrations in the liposome suspension.

## Data Availability

The data presented in this study are vailable on request from the corresponding author.

## Supplementary Materials

The following supporting information can be downloaded at: https://www.mdpi.com/article/10.3390/ijms232112817/s1.
