# Supplementary material for: Dual-Functionalized Nanoliposomes Achieve a Synergistic Chemo-Phototherapeutic Effect
Source: Int J Mol Sci. 2022 Oct 24;23(21):12817. doi: 10.3390/ijms232112817 (PMC9653560; doi:10.3390/ijms232112817)
Supplement: Supplementary file 1 [file ijms-23-12817-s001.zip › ijms-1971672-supplementary.pdf]

Electronic Supporting Information (ESI)

Dual-functionalized nanoliposomes achieve a synergistic chemo-phototherapeutic effect

Ana Lazaro-Carrillo, Beatriz Rodríguez-Amigo, Margarita Mora, M. Lluïsa Sagristá, Magdalena Cañete, Santi Nonell * and Angeles Villanueva *

Supplementary information: Figure S1: Cytotoxicity of empty DSPC:DOPS:CHOL liposomes, $CPT11_{lip}$ and $PpIX_{lip}$ as a function of concentration: Determination of IC50. Figure S2: Absorption and fluorescence emission spectra of CPT11 and PpIX solutions, in lactate buffer at pH 4.4 and THF, respectively. Figure S3: Size (Z-average mean) evolution over the time of the bimodal liposome formulation ($CPT11\text{-}PpIX_{lip}$). Figure S4: Fluorescence emission spectra of $CPT11\text{-}PpIX_{lip}$ showing the CPT11 and PpIX spectral region. Spectra of free drugs in solution are given for comparison. Figure S5: Time-Resolved Fluorescence of $CPT11\text{-}PpIX_{lip}$ suspensions in lactate buffer, showing CPT11 and PpIX spectroscopic region. Figure S6: Time-resolved $^1O_2$ phosphorescence signals in buffered aqueous suspensions of bimodal liposomes, $CPT11\text{-}PpIX_{lip}$. Table S1: Fluorescence quantum yields and fluorescence lifetimes. Table S2: Evaluation of photocytotoxicity of the bimodal treatment with $CPT11\text{-}PpIX_{lip}$, according to the Valeriote and Lin method. Video S1: Videomicroscopy analysis of control (untreated) cells; Video S2: Videomicroscopy study of HeLa cells treated with $CPT11_{lip}$; Video S3: Time lapse (24 h) videomicroscopy of HeLa cells after incubation with $PpIX_{lip}$ and irradiation; Video S4: Time-lapse videomicroscopy of HeLa cells treated with $CPT11\text{-}PpIX_{lip}$ and irradiated.

Characterization of bimodal nanoliposomes (CPT11-PpIX$_{lip}$)

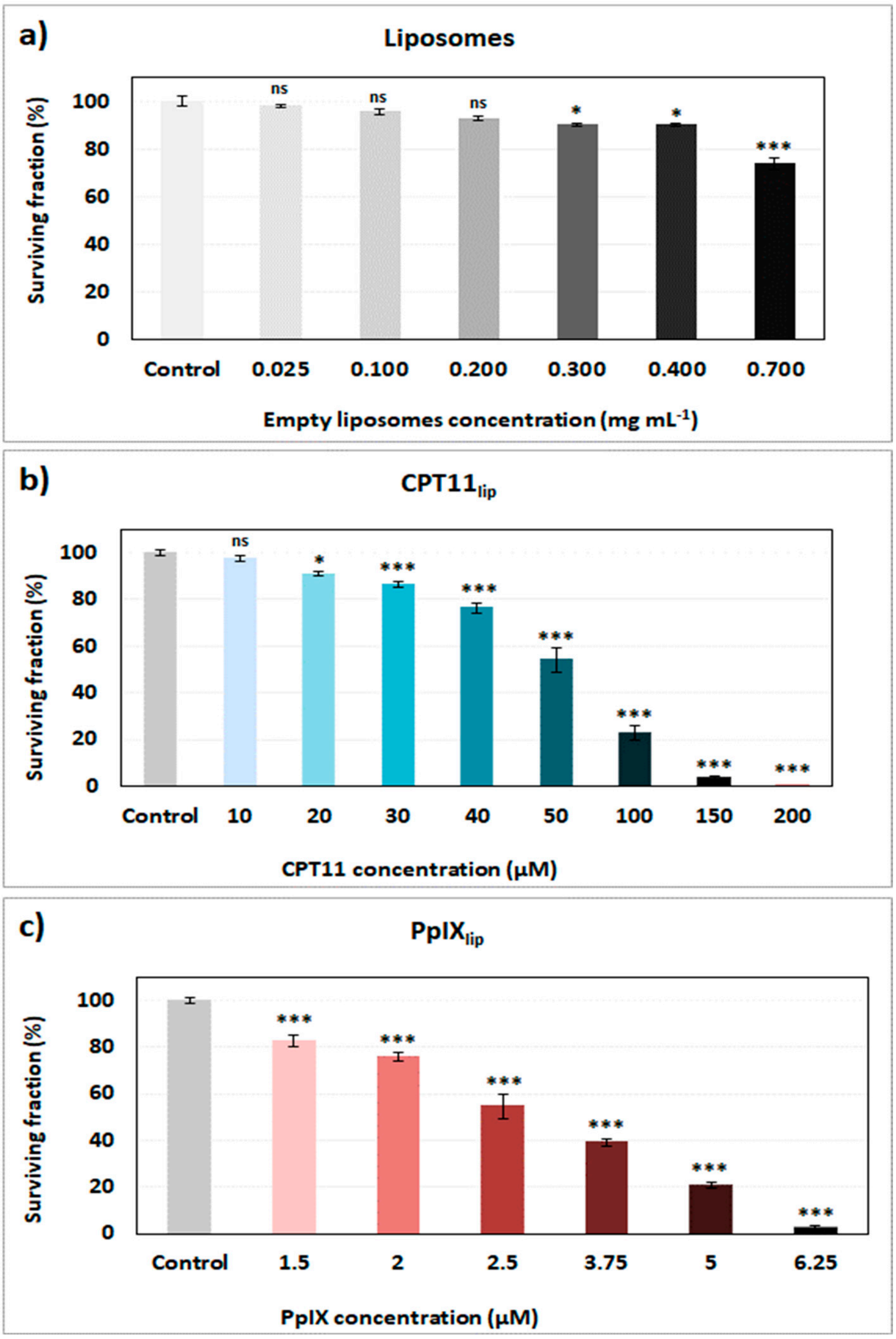

Figure S1. Cytotoxicity of the liposomes nanoformulations as a function of concentration. The cell viability was evaluated by the MTT assay performed 24 h after 24 h incubation with different amounts of **(a)** blank DSPC:DOPS:CHOL liposomes, **(b)** CPT11$_{lip}$, or **(c)** PpIX$_{lip}$ irradiated at 2 J cm^{-2}. Concentration of lipid in figures b and c was in the range of 0.054-1.08 mg mL^{-1} and 0.160-0.690 mg mL^{-1}, respectively. The estimated IC50 values for CPT11 and PpIX, when encapsulated in liposomes, from figures b and c were 53.3 µM (about 50 µM) and 2.8 µM (about 2.5 µM), respectively.

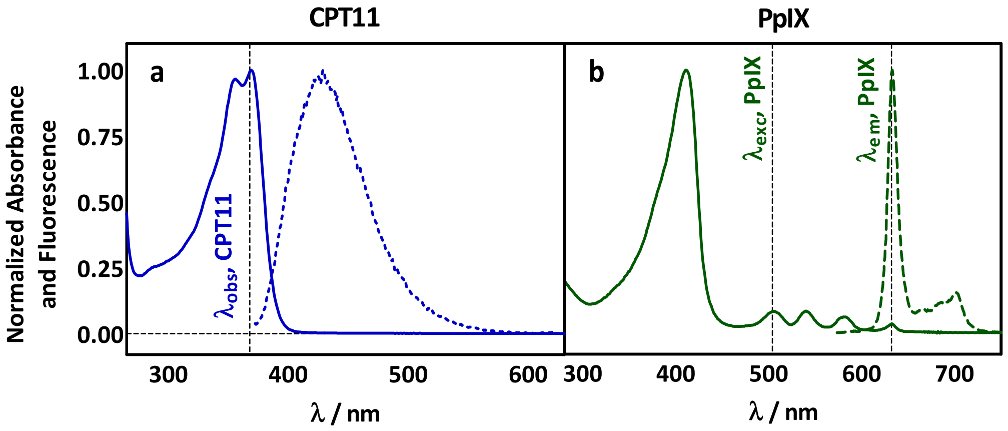

Figure S2. Absorption (solid line) and fluorescence emission (dashed line) spectra of CPT11 (blue) and PpIX (green) solutions, in lactate buffer at pH 4.4 and THF, respectively. In vertical dashed lines are pointed the wavelengths used for the measurements of the entrapment efficiencies either for CPT11 or PpIX.

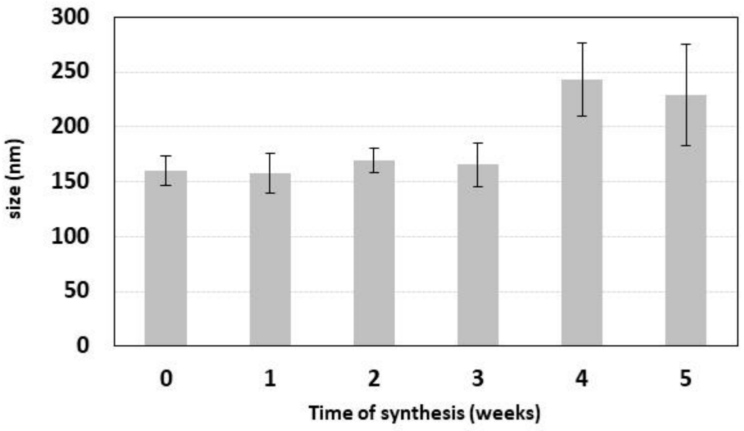

Figure S3. Time evolution of the bimodal liposomes' (CPT11-PpIX$_{lip}$) size (Z-average mean).

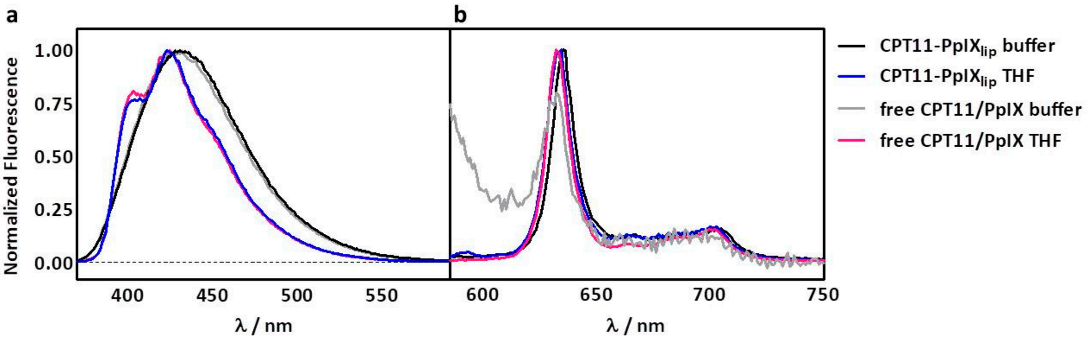

Figure S4. Fluorescence emission spectra of the bimodal liposomes CPT11-PpIX$_{lip}$, showing the CPT11 **(a)** and PpIX **(b)** spectral region. CPT11-PpIX$_{lip}$ suspensions were diluted in lactate buffer (pH 4.4) or in THF. Spectra of free drugs in solution are given for comparison. The λ_{exc} for CPT11 and PpIX were set at 360 nm and at 504 nm, respectively. Spectra of free drugs in solution are given for comparison

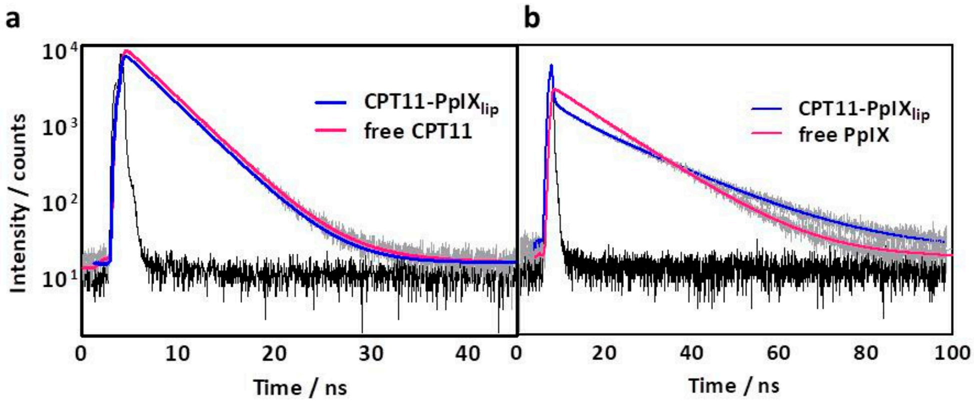

Figure S5. Time-resolved fluorescence decays of the bimodal liposomes CPT11-PpIX$_{lip}$ in lactate buffer (pH 4.4). Panel **(a)** corresponds to the CPT11 decay (λexc 375 nm, λobs 430 nm). Panel (**b**) corresponds to the PpIX decay (λexc 502 nm, λobs 700 nm). Time-resolved fluorescence decays of free drugs in solution are given for comparison. The black line corresponds to instrumental response factor (IRF).

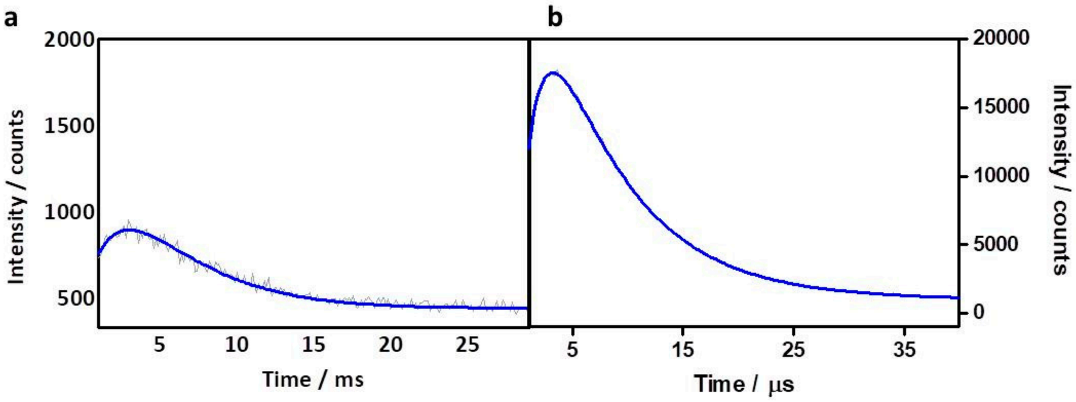

Figure S6. Time-resolved $^1O_2$ phosphorescence signals photosensitized by the bimodal liposomes CPT11-$PpIX_{lip}$ in air-saturated buffered aqueous suspensions. Panel (a) corresponds to $^1O_2$ photosensitized by CPT11 (λexc 355 nm, λobs 1275 nm). Panel (b) corresponds to $^1O_2$ photosensitized by PpIX (λexc 532 nm, λobs 1275 nm)

Table S1. Fluorescence quantum yields, lifetimes, and relative amplitudes of CPT11 and PpIX in the bimodal liposomes CPT11-$PpIX_{lip}$ in lactate buffer pH 4.4. The respective values for both drugs free in solution are also presented.

Sample	Solvent	λ_{exc} (λ_{obs})/ nm	τ_1 / ns (A%)	τ_2 / ns (A%)	Φ_F
free CPT11	buffer	375 (430)	3.52 (99.9%)	24.71 (0.1%)	0.62 ± 0.01
CPT11-$PpIX_{lip}$	buffer		3.54 ± 0.02 (100%)	-	0.38 ± 0.06
free PpIX	THF	502 (700)	11.61 (100%)	-	0.126 ± 0.004
CPT11-$PpIX_{lip}$	buffer		16.20 ± 0.53 (71.3%)	4.57 ± 0.30(28.7%)	0.19 ± 0.05

Table S2. Photocytotoxicity of the bimodal liposomes CPT11-$PpIX_{lip}$. Data in this table were obtained from the experimental results indicated in Figure 1 of the manuscript. The effect of photodynamic treatment with bimodal liposomes containing both drugs was evaluated at 24 h after the treatment, according to the Valeriote and Lin method, with: [A] representing cell viability for photodynamic treatment with $CPT11_{lip}$, [B] cell viability for photodynamic treatment with $PpIX_{lip}$, and [A + B] cell viability for photodynamic treatment with bimodal liposomes CPT11-$PpIX_{lip}$. Combined effects were defined as follows: synergistic [A + B] < [A] x [B] / 100 and subadditive [A] x [B] / 100 < [A + B] < [A], if [A] < [B].

Light dose (J.cm^{-2})	[A + B]	[A] · [B] / 100	Type of effect
0	56.4 ± 2.3	52.6 ± 7.8	Subadditive
2	12.6 ± 2.7	28.5 ± 3.0	Synergistic

Supplementary videos. HeLa cells seeded in chambered coverslip (Ibidi; Martinsried, Germany) were recorded by videomicroscopy after the different treatments. Frames were acquired by phase contrast microscopy every 15 min from 0 to 24 h after irradiation, maintaining CO_2, temperature and humidity conditions in cell culture range.

Supplementary video S1. Videomicroscopy analysis of control (untreated) cells.

Supplementary video S2. Videomicroscopy study of HeLa cells treated with $CPT11_{lip}$.

Supplementary video S3. Time lapse (24 h) videomicroscopy of HeLa cells after incubation with $PpIX_{lip}$ and irradiation.

Supplementary video S4. Time-lapse videomicroscopy of HeLa cells treated with bimodal $CPT11\text{-}PpIX_{lip}$ and irradiated.
